# To embrace or to avoid: the dual path effects of digital technology requirements on employees with different digital literacy

**DOI:** 10.3389/fpsyg.2025.1676932

**Published:** 2026-01-12

**Authors:** Shuang Li, Yumei Wang, Xiao Wang

**Affiliations:** 1 College of Digital Economics & Management, Mianyang Teachers’ College, Mianyang, China; 2 School of Business Administration, Southwestern University of Finance and Economics, Chengdu, China

**Keywords:** challenge stress, digital literacy, enterprise digital technology requirements, hindrance stress, proactive behavior, the Cognitive Appraisal Theory of Stress, turnover intention

## Abstract

Whether and how enterprise digital technology requirements affect employees constitutes a pivotal scholarly inquiry in the digital economy landscape. Grounded in the Cognitive Appraisal Theory of Stress and integrating the Job Demands-Resources (JD-R) Model with the Conservation of Resources (CoR) Theory, this study proposes employees may appraise enterprise digital technology requirements as either challenge stressors-proactively embracing demands to catalyze positive outcomes in proactive behaviors-or hindrance stressors-cognitively avoiding requirements thus eliciting negative consequences in turnover intention. Moreover, employees’ digital literacy levels fundamentally reconfigure their cognitive appraisals of these technological demands, thereby triggering differential behavioral cascades. Utilizing a three-wave longitudinal survey design with 332 validated responses from employees undergoing digital transformation, this study employed AMOS 23.0 for confirmatory factor analysis, SPSS 23.0 for hierarchical regression modeling, and the PROCESS macro for bootstrap mediation tests to substantiate the proposed theoretical framework. This study advances the dual-path framework of enterprise digital technology implementation by elucidating how digital literacy-as a personal resource-shapes divergent outcomes stemming from organizational digital demands. Furthermore, it extends the application of the Cognitive Appraisal Theory of Stress to digital contexts through empirical validation, and also to provide some inspiration and reference for relevant management practices.

## Introduction

Against the backdrop of the global economy’s accelerating shift toward digitalization and intelligence, the significance of the digital economy has become increasingly prominent. In 2023, China’s digital economy reached a scale of RMB 53.9 trillion, contributing 41.6% to the nation’s GDP (China Academy of Information and Communications Technology, 2024). Consequently, policy makers in China have been continuously strengthening strategic support for digital transformation. China’s *14th Five-Year Plan for Digital Economic Development* advocates for accelerating enterprise digital transformation to enhance supply chain collaboration efficiency and strengthen economic resilience. Concurrently, The Six Pillars of Educational Digital Transformation underscores “coordination and leadership” as a core pillar, emphasizing the need for a systemic framework to guide organizations in adapting to digital disruption. These policy directives demonstrate that digital transformation has become a critical pathway for enterprises to bolster their core competitiveness. Digital transformation not only reshapes the technological infrastructure of enterprises but also imposes new demands on organizational structures, culture, and employee competencies. Existing research indicates that corporate digital transformation alters employees’ job content, work patterns, and processes, further generating new job demands (e.g., skill specifications). These are formally termed by scholars as enterprise digital technology requirements (Fu et al., 2024).

Organizations introduce digital technology and undertake digital transformation with the primary objective of optimizing productivity and enhancing production quality (Jarrahi, 2018). In practice, however, digital technology application in enterprises has yielded mixed evaluations. From a positive perspective, digital technology application in enterprises may enhance creativity (Wang et al., 2022; Ouyang and Zhu, 2025), thereby stimulating innovative behaviors (Fu et al., 2024; Wang et al., 2024), promoting employees’ proactive learning behaviors (Zou et al., 2023), and boosting well-being perceptions (Loureiro et al., 2023). Corporate digital transformation exerts a significant positive impact on employees’ proactive assumption of work responsibilities through the mediating roles of work autonomy, self-efficacy, and closeness to colleagues (Liu and Cheng, 2025). Conversely, digital technology application may trigger perceived emotional exhaustion (Wang et al., 2024), job burnout (Meng and Xu, 2024), and work insecurity (Johnson et al., 2020), consequently inducing deviant behaviors (Xu, 2023) and knowledge hiding behaviors (He C. et al., 2024). Simultaneously, digital technology application may yield divergent outcomes across individuals and organizations. For instance, employees’ differential perceptions of enterprise digital technology requirements can trigger both positive and negative consequences (He Q. et al., 2024; Liang et al., 2022; Liu et al., 2024; Zhang et al., 2023). Distinct features of digital technology requirements exert diametrically opposed effects on employees’ innovative behaviors (Verma and Singh, 2022). For employees with varying skill sets, digital technology application differentially impacts their creativity (Jia et al., 2024). Among individuals performing divergent job tasks, such technologies may enhance or diminish work performance (Tong et al., 2021). Compulsory versus voluntary adoption further generates dual effects on employee creativity (Li and Guo, 2025). For employees with internal and external locus of control, their evaluations of the challenging and hindering aspects of artificial intelligence technology adopted by enterprises differ (Cheng et al., 2023). For employees with varying levels of engagement within an enterprise, artificial intelligence job demands may induce stress that affects their well-being, but may also serve as motivating factors that stimulate work motivation (Loureiro et al., 2023). Research on the impact of digital technology application on organizational behaviors has garnered significant scholarly attention (Bankins et al., 2024). A growing number of scholars recognize that enterprise digital adoption is not a “binary phenomenon,” but rather yields divergent effects contingent on contextual factors. Building on this logic, the present study employs the Cognitive Appraisal Theory of Stress (Lazarus and Folkman, 1987) to examine how employees’ differential cognitive appraisals of digital technology requirements may trigger dichotomous outcomes: embracing versus avoidance responses. Crucially, variations in employees’ digital literacy are posited to moderate these dual effects.

The Cognitive Appraisal Theory of Stress posits that individuals cognitively appraise stressors as either challenges or threats based on their perceived resources, triggering corresponding coping behaviors (Lazarus and Folkman, 1987). As a form of job demand, digital technology requirements constitute an occupational stressor that prompts employees’ stress responses in the workplace (Brougham and Haar, 2020). According to the Cognitive Appraisal Theory of Stress (Lazarus and Folkman, 1987), employees may cognitively appraise digital technology requirements as either challenges or threats. Those who develop challenge appraisals engage in skill enhancement and competency development through proactive efforts, thereby implementing proactive work behaviors. This adaptive process enables them to rapidly acclimate to and actively embrace the digital transformation context (Wang et al., 2024). Individuals who form threatening appraisals – perceiving digital technology requirements as impediments-may exhibit avoidance behaviors and even develop turnover intentions (Jianwu et al., 2024). Existing studies, focusing on the stress induced by digital technology itself, have confirmed that technostress can be appraised either as a challenge technostress, leading to positive outcomes such as enhanced work flexibility, increased overall job engagement, and a stronger sense of work autonomy, or as a threat technostress, resulting in avoidance behaviors (Tarafdar et al., 2019; Tarafdar et al., 2020). Based on the JD-R model (Demerouti et al., 2001), which categorizes job demands using a “dichotomous” approach to stressors, (Van Den Broeck et al., 2010) found that certain job demands-such as workload and cognitive demands-function as challenge stressors, promoting a challenge appraisal and subsequently generating positive effects. Conversely, other job demands-like work–family conflict and emotional demands-act as hindrance stressors, leading to a hindrance appraisal and thus producing negative outcomes. The corporate digital technology requirements examined in this study may exhibit characteristics of both workload and cognitive demands, thereby fostering challenge appraisal and positive effects (Van Den Broeck et al., 2010), while also potentially sharing traits with work–family conflict and emotional demands, thus promoting hindrance appraisal and negative consequences (Van Den Broeck et al., 2010). Consequently, this study integrates challenge-hindrance stress into its theoretical framework to examine dual-path mediating effects through which digital technology requirements influence employees’ proactive behaviors (embracing) and turnover intentions (avoidance).

Building on the Cognitive Appraisal Theory of Stress, individuals’ cognitive appraisals depend on their perceived capacity to mobilize resources when confronting stressors (Li and Chen, 2024). Digital literacy encompasses an individual’s ability to use digital technologies and devices, as well as related affective, learning, and cognitive capacities (Matarazzo et al., 2021). It comprises three dimensions: knowledge, skills, and attitudes (Makowska-Tłomak et al., 2023), serving as a critical resource that influences how individuals cognitively appraise stressors such as enterprise digital technology demands. Individuals with high digital literacy adeptly navigate digital tools and systems. Drawing on self-efficacy theory (Bandura, 1989), when confronting digital technology requirements, they respond confidently and proactively, exhibiting a greater propensity for challenge appraisals (Wang and Liu, 2025). Conversely, those with limited digital literacy often become overwhelmed by difficulties and anxiety induced by such requirements, leading to heightened threat appraisals. Furthermore, integrating the JD-R model with the COR Theory provides a dual theoretical lens. On one hand, the “buffering” hypothesis from the JD-R model posits that job resources can mitigate the depleting effects of high job demands, thereby alleviating their negative impact on employees (Bakker et al., 2004). On the other hand, job demands may pose a threat of resource loss for employees. According to the resource investment principle of COR theory, individuals invest resources to protect their existing resource pool from further depletion (Halbesleben et al., 2014). It can thus be inferred that the investment of a resource such as digital literacy can foster a more positive appraisal of job demands like digital technology requirements (i.e., promoting challenge appraisal) while reducing their negative appraisal (i.e., diminishing hindrance appraisal). Therefore, this study introduces digital literacy as a moderator to investigate its potential moderating role in the impact mechanisms of digital technology requirements on employees’ proactive behaviors and turnover intentions.

**Figure 1:**
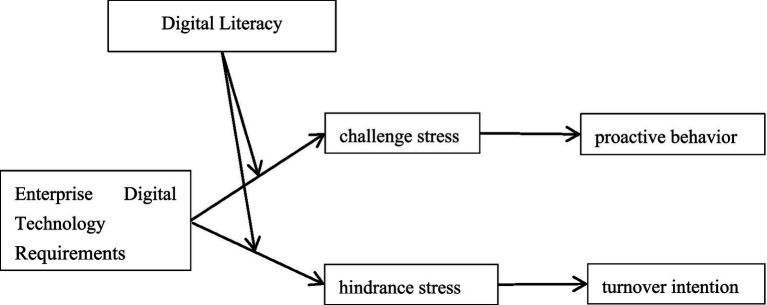
Proposed theoretical model.

Collectively grounded in the Cognitive Appraisal Theory of Stress and integrating the JD-R Model with the COR Theory, following the logic of subjective stress experience and the adequacy of coping resources (Makowska-Tłomak et al., 2023), this study specifically examines how digital technology requirements influence proactive employee behaviors via challenge stress-moderated by digital literacy-and concurrently investigates their impact on turnover intentions through hindrance stress under digital literacy’s moderating role, as theorized in Figure 1. This study primarily addresses three research questions: First, how do digital technology requirements affect employees’ proactive behaviors and turnover intentions, and is this impact mediated by challenge stress and hindrance stress? Second, do the effects of digital technology requirements on challenge stress and hindrance stress vary across different levels of digital literacy? Third, does digital literacy moderate the pathways through which digital technology requirements influence proactive behaviors and turnover intentions via distinct stress appraisals? Addressing these questions will advance our understanding of the dual-path mechanisms underlying the double-edged effects of digital technology application, reveal how digital literacy moderates the consequences of digital technology requirements, and deepen the application of the Cognitive Appraisal Theory of Stress in digital contexts.

## Theory and hypotheses

### Cognitive Appraisal Theory of Stress

The Cognitive Appraisal Theory of Stress was proposed by Lazarus and Folkman (1984). Its core proposition asserts that variations in stress responses stem from individuals’ differential cognitive appraisals of stressors, rather than direct stimulation by the stressors themselves (Lazarus and Folkman, 1984). When confronting a stressor, individuals first engage in primary appraisal-an initial assessment of the stressor’s potential consequences. This appraisal typically manifests as either a challenge appraisal or a threat appraisal. The former occurs when individuals perceive the stressor as conducive to goal achievement and personal growth, representing a reward and opportunity; the latter arises when they view the stressor as detrimental to goals and development, constituting an obstacle and threat (Du and Chen, 2023). Following primary appraisal of a stressor, individuals proceed to secondary appraisal-the evaluation of coping strategies based on available resources and capabilities. Those with challenge appraisals adopt proactive problem-focused strategies, actively embracing challenges, whereas individuals making threat appraisals employ avoidant emotion-focused strategies (Zhou et al., 2023). The Cognitive Appraisal Theory of Stress further posits that individuals engage in differential cognitive appraisals of external stressors based on their personal conditions-including available resources, capabilities, and individual characteristics (Lazarus and Folkman, 1984).

### Main effects pathway: the impact of digital technology requirements on employees’ proactive behaviors and turnover intentions

Following the pervasive integration of digital technologies across traditional and emerging industries, significant transformations have occurred in job content and business processes, consequently imposing heightened digital technology requirements on employees (Zhu et al., 2020). Such requirements exhibit a dual nature-functioning as both a stressor and a stimulus (Fu et al., 2024). On one hand, digital technology requirements may stimulate employees’ intrinsic vigor, thereby motivating proactive work efforts. On the other hand, they may drain substantial energy resources, inducing heightened psychological strain that ultimately fosters work disengagement or aversion (Gonzalez-Mulé et al., 2021; Huth and Chung-Yan, 2022).

“Embracing”: The positive pathway through which digital technology requirements facilitate employees’ proactive behaviors.

Employees’ proactive behaviors refer to self-initiated actions through which individuals anticipate, initiate, and respond to changes in work systems or roles (Griffin et al., 2007). These behaviors entail employees spontaneously proactively anticipating potential problems, needs, or transformations before their materialization, devising solutions, and taking preemptive actions to catalyze change—rather than reactively responding post-occurrence (Parker and Collins, 2010). Consequently, such behaviors assume heightened significance against the backdrop of VUCA (Volatility, Uncertainty, Complexity, Ambiguity) environments amplified by global digitalization (Griffin et al., 2007). Empirical evidence indicates that job characteristics-such as autonomy and work enrichment-constitute pivotal contextual factors significantly shaping employees’ proactive behaviors, alongside individual attributes (Kong and Li, 2019). Correspondingly, management practices effectively stimulate such behaviors by redesigning work features-for instance, through expanding decision latitude and enriching task content (Zhao and Zhai, 2018; Wei and Pan, 2012). Critically, the routine adoption of enterprise digital technologies serves as an effective mechanism for modifying these characteristics by enhancing job autonomy and work enrichment. In organizational contexts, the application of digital technologies is defined as the utilization of internet, big data, artificial intelligence, IoT, and related systems across workplace domains-including administration, production, operations, and R&D (Piccinini et al., 2015). These technologies potentially grant employees heightened flexibility and autonomy, enabling conditional self-determination in work location/scheduling while reducing excessive supervisory oversight and in-person interruptions. Consequently, employees achieve maximal self-management and autonomous work execution (Wang and Du, 2021), thereby enhancing perceived job control and reinforcing their belief in mastering work through proactive behaviors. Simultaneously, such autonomy confers additional psychological resources-a metaphorical energy reservoir that facilitates the implementation of proactive initiatives (Xu et al., 2024).

The application of digital technology in enterprises gives rise to novel work scenarios and tasks, necessitating employees’ proactive adoption of innovative cognitive frameworks and methodologies to address emerging challenges. Simultaneously, empowered by identifying digital opportunities, employees proactively employ new tools and pathways to resolve conventional work problems (Fu et al., 2024). The open architecture and interconnectivity of digital technologies enhance data accessibility and resource availability (Smith et al., 2017). Through digital technology application, employees gain empowered access to extract knowledge, information, and material resources (Ma, 2024), thereby fostering more conducive conditions for the enactment of employees’ proactive behaviors. Digital infrastructure-including components and platforms-provides employees with streamlined access to knowledge acquisition and developmental training, facilitating career advancement and skill enhancement (Wang and Du, 2021). This empowerment thereby motivates heightened engagement in proactive behaviors to fulfill work responsibilities more effectively. Existing studies based on the Technology Acceptance Model have confirmed that prolonged interaction with technology may contribute to the development of new proactive behaviors. The Job Demands-Resources model has also demonstrated that job demands can promote motivational processes through positive outcomes (Ingusci et al., 2021). In summary, the following hypothesis is proposed:

*H1:* Digital technology requirements positively influence employees' proactive behaviors.

“Avoidance”: The negative pathway through which digital technology requirements facilitate employees’ turnover intentions.

Turnover intentions refer to employees’ deliberate plans to discontinue membership with their current organization (Tett and Meyer, 1993). Empirical evidence consistently identifies job demands as critical antecedents of turnover intentions (Azharudeen and Arulrajah, 2018). Crucially, digital technology requirements impose heightened novel demands on employees, eliciting significant psychological strain. This strain manifests as work disengagement behaviors-diminished organizational contributions-which subsequently foster stronger turnover intentions (Teng et al., 2024). Moreover, while the routine adoption of digital technology requirements expands job autonomy, it concurrently establishes pervasive accessibility-enabling 24/7 work connectivity. This constant availability precipitate’s role overload and emotional exhaustion (Park et al., 2020). Consequently, employees develop a perceived loss of autonomy and psychological deprivation, ultimately triggering work disengagement and heightened turnover intentions (Yu et al., 2024). Furthermore, digital technology application may induce employees’ perceived undervaluation of personal worth or eroded self-efficacy (Presbitero and Teng-Calleja, 2023), exacerbate job insecurity (Li et al., 2019), and deplete psychological resources (Jianwu et al., 2024). Collectively, these states amplify the pronounced manifestation of turnover intentions (Raj and Seamans, 2019). The “creative destruction” inherent in digital technologies is reshaping labor market structures by displacing traditional occupations and transforming job characteristics, thereby optimizing resource allocation. This process, to some extent, intensifies employees’ pessimistic career prospects within organizations (Guo and Wu, 2024), ultimately fostering turnover intentions.

The JD-R model posits that when job demands exceed available resources, it triggers a progressive health impairment process leading to negative consequences (Ingusci et al., 2021). The Cognitive Appraisal Theory of Stress posits that when confronted with overwhelming stressors exceeding coping capacities, individuals adopt avoidance or detachment strategies to mitigate impacts (Ma and Li, 2025). Within this framework, turnover intentions constitute one such behavioral mechanism. Consequently, the following hypothesis is proposed:

*H2:* Digital technology requirements positively influence employees' turnover intentions.

### Mediating pathways: the intermediary role of challenge stress and hindrance stress

Challenge stress denotes pressure that evokes individuals’ motivational drive and positive activation, perceived as conducive to goal attainment. Such stress typically stems from demanding yet developmental stressors like heavy workloads, time urgency, and heightened responsibilities (Cavanaugh et al., 2000). Crucially, challenge stress yields beneficial work outcomes, including elevated job satisfaction, enhanced task performance, and increased organizational citizenship behaviors. Hindrance stress constitutes pressure diametrically opposed to challenge stress, characterized by imposed constraints that frustrate goal progress and impede the attainment of valued objectives. Such stress typically stems from developmentally obstructive stressors like organizational politics and job insecurity (Cavanaugh et al., 2000), culminating in adverse work outcomes including job dissatisfaction and voluntary turnover.

Primarily, as a form of job demand, digital technology requirements may simultaneously elevate challenge stress and intensify hindrance stress among employees. Stressors exhibit a dual nature, categorized into challenge stressors (work challenges) and hindrance stressors (work impediments). Challenge stressors denote work conditions that ignite employees’ motivational drive, foster goal attainment, and facilitate personal growth; conversely, hindrance stressors constitute obstructive work characteristics that elicit negative affect, deplete energy reserves, and thwart goal achievement (Van Den Broeck et al., 2010). Building on the Cognitive Appraisal Theory of Stress, employees may appraise digital technology requirements as either challenge stressors or hindrance stressors. When enterprises deploy such technologies across administrative, production, and operational domains, employees may perceive enhanced work facilitation-liberating them from highly repetitive, routine, and structured tasks-thereby enabling the allocation of premium cognitive resources toward resolving complex mission-critical problems (Glikson and Woolley, 2020; Kożusznik et al., 2018). Concurrently, digital technology application empowers employees to acquire, process, and analyze expansive information and data repositories, thereby augmenting their functional capabilities (Glikson and Woolley, 2020; Kożusznik et al., 2018). These enhancements may be perceived as conditions conducive to goal attainment and personal growth, ultimately triggering challenge appraisals. Conversely, digital technology application necessitates substantial energy and resource investment from employees to adapt to altered work content and processes. Moreover, it may engender perceived job displacement risks, fostering job insecurity (Johnson et al., 2020). Furthermore, by reducing face-to-face interactions, such technologies can induce psychological isolation (Tang et al., 2023). Collectively, these conditions may be appraised as hindrance stressors-eliciting negative affect, depleting cognitive reserves, and impeding goal attainment-thereby precipitating hindrance appraisals. Consequently, the following hypotheses are proposed:

*H3a:* Digital technology requirements positively influence employees' challenge stress.

*H4a:* Digital technology requirements positively influence employees' hindrance stress.

Furthermore, challenge stress and hindrance stress, respectively, elicit employees’ proactive behaviors and turnover intentions. Empirically, extant research confirms that challenge stress positively influences work attitudes and behaviors, whereas hindrance stress elicits contrary effects (Li and Li, 2013). When experiencing challenge stress, employees perceive that overcoming such challenges aligns with organizational expectations and yields commensurate rewards conducive to personal growth (May et al., 2004). Consequently, they proactively mobilize their motivation, exercise subjective agency within organizational contexts, and activate proactive behaviors to resolve work-related problems (Cavanaugh et al., 2000). Conversely, when confronted with hindrance stress, employees experience helplessness, anxiety, and work-related fatigue (Crawford et al., 2010). Consequently, they adopt work avoidance and disengagement strategies (Lazarus and Folkman, 1987). Empirical evidence substantiates that hindrance stress directly elevates employees’ turnover intentions (Boswell et al., 2004). Digital transformation pressure constitutes a hindrance stressor that leads to employee work disengagement and subsequently fosters turnover intention (Makowska-Tłomak et al., 2022). Based on the above, the following hypotheses are proposed:

*H3b:* Challenge stress has a positive effect on employees' proactive behavior.

*H4b:* Hindrance stress has a positive effect on employees' turnover intention

Finally, according to the core tenets of the Cognitive Appraisal Theory of Stress (Lazarus and Folkman, 1984) and the JD-R model, individuals may develop two distinct cognitive appraisals-challenge assessment and hindrance assessment-when confronting stressors, subsequently adopting corresponding coping strategies. Individuals who develop challenge assessments are likely to trigger proactive behavioral responses to cope with stressors, whereas those forming hindrance assessments tend to exhibit avoidance-oriented behaviors (Zhou et al., 2023). Following this logic, it can be argued that digital technology requirements-as a workplace stressor-may trigger employees’ challenge or hindrance appraisals. Individuals forming challenge appraisals are likely to demonstrate proactive behavior, whereas those developing hindrance appraisals may exhibit elevated turnover intention. Building on H3a, H4a, H3b, and H4b, the following mediating hypotheses are proposed:

*H3:* Challenge stress mediates the relationship between digital technology requirements and employees’ proactive behavior.

*H4:* Hindrance stress mediates the relationship between digital technology requirements and employees’ turnover intention.

### The moderating role of digital literacy

The Cognitive Appraisal Theory of Stress further posits that an employee’s appraisal of stressors emerges from dynamic interactions with individual characteristics (e.g., personal resources, capabilities) (Lazarus and Folkman, 1984). The JD-R model posits that employees can reshape their jobs by increasing structural job resources (e.g., enhancing their own skills and decision-making influence), thereby stimulating proactive behaviors (Ingusci et al., 2021). For instance, existing research has found that highly-skilled employees, when empowered by AI, can develop positive emotional evaluations, which in turn predispose them to proactive job crafting (Jia et al., 2024). Job crafting g is defined as the self-initiated changes that employees make in their own job demands and job resources to attain and/or optimize their personal (work) goals, and it constitutes part of the proactive behaviors examined in this study (Tims et al., 2012). Conversely, low-skilled employees tend to form negative emotional evaluations when confronted with AI, further inclining them toward a passive approach to work design (Jia et al., 2024). Digital literacy refers to an individual’s capability to utilize digital technologies, which significantly enhances information acquisition capacities and facilitates effective access to digital resources-thereby enabling rapid adaptation to the digital society and work activities (Li et al., 2022). Variations in employees’ digital literacy substantially determine their engagement levels and benefit gains during organizational digital enablement processes (Su et al., 2021). Therefore, this study incorporates digital literacy as a critical individual characteristic into the theoretical model, examining how it may influence employees’ appraisals of digital technology requirements as a stressor and subsequently shape their behavioral responses.

High digital literacy may be associated with the appraisal of challenge stress, as individuals with advanced digital competencies are capable of effectively enhancing their coping abilities through effort when enterprises introduce digital technologies (Makowska-Tłomak et al., 2022), subsequently generating valuable outcomes (Lepine et al., 2005). Based on the buffering hypothesis of the JD-R model, resources can mitigate the negative impact of high job demands (Bakker et al., 2004). When integrated with the resource investment principle of the COR theory, which suggests that job demands may threaten resource depletion, employees invest existing resources to prevent the loss of their resource pool (Halbesleben et al., 2014). It follows that a resource such as high digital literacy helps buffer the negative effects posed by corporate digital technology requirements, while also enabling employees to invest this resource to counteract potential resource loss resulting from these demands. Therefore, individuals with high digital literacy possess sufficient agency to navigate novel organizational digital environments, where they can enact actions to attain valued outcomes (Mueller and Thomas, 2001). When confronting problems or difficulties, such individuals are more likely to recognize embedded opportunities for gain, thereby developing positive cognitive appraisals and adopting proactive coping strategies (Lepine et al., 2005). When organizations implement digital technologies for transformation, employees with high digital literacy are more likely to attain substantial career growth and success in this context. Thus, such individuals appraise digital technology requirements as challenge appraisals rather than hindrances (Cheng et al., 2023). Conversely, low digital literacy may associate with hindrance appraisals as individuals with deficient digital capabilities fail to generate valued outcomes through personal effort when organizations deploy digital technologies (Lepine et al., 2005). Specifically, individuals with deficient digital literacy lack the capabilities to navigate novel organizational digital environments and perceive such transformations as potential impediments to personal growth and development (Lepine et al., 2005). When organizations deploy digital technologies for transformation, employees with low digital literacy develop technological displacement concerns. Consequently, they interpret digital technology requirements as obstacles to their career advancement, ultimately appraising them as hindrance appraisals rather than challenges (Cheng et al., 2023). During primary appraisal, digital technology requirements elicit both challenge appraisals and hindrance appraisals among employees-a process moderated by individual digital literacy levels. Subsequently, employees engage in secondary appraisal to implement coping strategies (Lazarus and Folkman, 1984). Individuals forming challenge appraisals adopt approach-oriented coping strategies that facilitate self-development, whereas those developing hindrance appraisals employ avoidance-focused coping responses (Zhang et al., 2019). Based on the integrated theoretical framework, this study posits that individuals with high digital literacy are predisposed to activate challenge stress, thereby triggering proactive behavior, whereas those with low digital literacy tend to elicit hindrance stress, consequently manifesting heightened turnover intention. The following hypotheses are therefore proposed:

*H5a:* Digital literacy positively moderates the positive effect of digital technology requirements on employees' challenge stress. Specifically, this positive effect is stronger when employees possess higher levels of digital literacy.

*H5b:* Digital literacy positively moderates the indirect relationship between digital technology requirements and proactive behavior through challenge stress. Specifically, the indirect effect of digital technology requirements on proactive behavior via challenge stress is stronger when digital literacy is higher, and weaker when lower.

*H6a:* Digital literacy negatively moderates the positive effect of digital technology requirements on employees' hindrance stress. Specifically, this positive effect is weaker when employees possess higher levels of digital literacy.

*H6b:* Digital literacy negatively moderates the indirect relationship between digital technology requirements and turnover intention through hindrance stress. Specifically, the indirect effect of digital technology requirements on turnover intention via hindrance stress is weaker when digital literacy is higher, and stronger when lower.

## Methods

### Sample and process

This study employed an online survey methodology, recruiting participants from multiple enterprises across Sichuan, Beijing, Shanghai, and Jiangsu provinces. All participating organizations were required to be either currently undergoing or having completed digital transformation. To effectively mitigate common method bias, survey data were collected across three temporally separated waves at one-month intervals. The specific procedure entailed: (1) obtaining participant names/IDs to assign unique survey codes; (2) during Wave 1 (T1), collecting data on digital technology requirements, digital literacy, and demographic variables (432 questionnaires distributed, 398 valid responses retained, 92.1% valid response rate); (3) during Wave 2 (T2), assessing challenge stress and hindrance stress by readministering surveys to the 398 T1 participants using pre-assigned codes (398 questionnaires readministered, 352 valid responses retrieved, 88.4% retention rate); (4) during Wave 3 (T3), evaluating proactive behavior and turnover intention through identical read ministration to the 352 T2 respondents (352 questionnaires readministered, 332 valid responses retrieved, 94.3% retention rate).

Among the 332 valid responses, the sample characteristics were: 61.14% female; 87.35% aged 21–40; 89.15% holding a bachelor’s degree or higher; 37.3% with 1–5 years of work experience and 46.4% with 6–10 years; 60.24% employed in private enterprises versus 24.4% in state-owned enterprises; 28.61% in technical roles with the remainder (71.39%) in non-technical functions including management, marketing, finance, and production.

### Measures

This study exclusively employed well-established scales from prior literature, with all instruments subjected to *back-translation procedures* to ensure measurement validity and contextual adaptation for Chinese respondents (Richard, 1970). All questionnaire items (excluding demographic information) employed a 5-point Likert scale ranging from 1 (*strongly disagree*) to 5 (*strongly agree*). To ensure contextual fit, a pilot test was conducted with seven employees from one enterprise (excluded from the formal sample), followed by semantic refinements based on participant feedback-primarily enhancing item comprehensibility-before finalizing the survey instrument.

Digital technology requirements were measured using a 5-item scale adapted from Khin and Ho (2019), widely adopted by Chinese scholars. A sample item states: *“In my work, I need to access essential digital technologies.”* The scale demonstrated a Cronbach’s *α* of 0.856 in this study.

Challenge stress and hindrance stress were measured using scales developed by Cavanaugh et al. (2000). The former comprises six items (e.g., *“The sheer volume of projects/tasks I need to complete creates significant pressure”*; Cronbach’s *α* = 0.892), while the latter consists of five items (e.g., *“I experience feelings of job insecurity”*; Cronbach’s *α* = 0.873).

Proactive behavior was assessed using a 3-item scale developed by Griffin et al. (2007), widely validated in Chinese organizational contexts. A sample item reads: *“I initiate improved approaches to accomplish my core work responsibilities.”* The scale yielded a Cronbach’s *α* of 0.806.

Turnover intention was measured with a 4-item scale developed by Kelloway et al. (1999). A representative item states: *“I am actively considering leaving this organization.”* The scale demonstrated a Cronbach’s *α* of 0.83.

Digital literacy was assessed using a 10-item scale developed by Ng (2012). A sample item includes: *“I know how to troubleshoot my own technical issues.”* The scale achieved a Cronbach’s *α* of 0.938.

Control variables included employee gender, age, and educational attainment, following established practices in prior research, as these demographic factors significantly influence attitudes and behaviors during organizational change (Madsen et al., 2005).

## Results

### Common method variance test

To mitigate common method bias, this study implemented a three-wave survey design with three-month intervals and minimized participant apprehension through standardized instructions. Nevertheless, given the exclusive reliance on self-reported employee data, potential measurement bias inevitably persists and requires statistical verification. Consistent with established protocols, Harman’s single-factor test (Harman, 1976) was conducted. Exploratory factor analysis (EFA) of all 33 items revealed six factors with eigenvalues exceeding 1.0, with the primary factor accounting for 32.13% of the variance-significantly below the 50% threshold of the total variance explained (66.54%). These results indicate the absence of substantial common method bias (Malhotra et al., 2006; Podsakoff et al., 2003).

### Validity test

Given the utilization of well-established scales, confirmatory factor analysis (CFA) was performed via AMOS 23.0 to examine the discriminant validity of six latent variables: *digital technology requirements*, *digital literacy*, *challenge stress*, *hindrance stress*, *proactive behavior*, and *turnover intention*. Goodness-of-fit indices for all measurement models were comparatively assessed (as presented in Table 1). As evidenced in Table 1, the six-factor model demonstrated superior fit with the following indices: $\frac{\chi^{2}}{df}$ = 1.246, TLI = 0.979, CFI = 0.981, *RMSEA* = 0.027. Thus, the six measured variables demonstrate robust discriminant validity, confirming they represent distinct constructs. This finding further corroborates the absence of substantial common method bias in this study.

**Table 1:** Results for confirmatory factor analysis.

Model	*χ* ^2^	*df*	$\frac{\chi^{2}}{df}$	*RMSEA*	*RMR*	*TLI*	*CFI*
DR, DL, CS, HS, PB, TI	589.048	480	1.246	0.027	0.047	0.979	0.981
DR, DL, CS, HS, PB + TI	858.052	485	1.769	0.048	0.063	0.933	0.939
DR, DL, CS, HS + PB + TI	1,175.368	489	2.404	0.065	0.080	0.878	0.887
DR, DL, CS + HS + PB + TI	1,520.473	492	3.090	0.079	0.096	0.819	0.831
DR, DL + CS + HS + PB + TI	3,267.115	494	6.614	0.130	0.244	0.514	0.545
DR + DL + CS + HS + PB + TI	3,591.857	495	7.256	0.137	0.186	0.458	0.492

*N = 332; DR, digital technology requirements; DL, digital literacy; CS, challenge stress; HS, hindrance stress; PB, proactive behavior; TI, turnover intention.*

### Descriptive statistics

Using SPSS 23.0, descriptive statistics for key variables-including means (M), standard deviations (SD), and correlation coefficients-were computed and are detailed in Table 2. As shown in the table, digital technology requirements exhibit a statistically significant positive correlation with proactive behavior (*r* = 0.576, *p* < 0.01), digital technology requirements demonstrate a statistically significant positive correlation with turnover intention (*r* = 0.455, *p* < 0.01), digital technology requirements demonstrate a statistically significant positive correlation with challenge stress (*r* = 0.514, *p* < 0.01), digital technology requirements exhibit a statistically significant positive correlation with hindrance stress (*r* = 0.425, *p* < 0.01), challenge stress shows a statistically significant positive correlation with proactive behavior (*r* = 0.469, *p* < 0.01), hindrance stress manifests a statistically significant positive correlation with turnover intention (*r* = 0.504, *p* < 0.01), digital literacy evidences a statistically significant positive correlation with challenge stress (*r* = 0.302, *p* < 0.01). These findings provide preliminary empirical support for the theoretical relationships underpinning subsequent hypothesis testing.

**Table 2:** Means, standard deviations and correlation coefficients of variable.

Variables	M	SD	1	2	3	4	5	6
1. Digital technology requirements	3.905	0.759	1					
2. Digital literacy	3.856	0.847	0.288^**^	1				
3. Challenge stress	3.005	1.084	0.514^**^	0.302^**^	1			
4. Hindrance stress	2.781	0.998	0.425^**^	0.209^**^	0.504^**^	1		
5. Proactive behavior	4.016	0.734	0.576^**^	0.313^**^	0.469^**^	0.398^**^	1	
6. Turnover intention	2.499	1.064	0.455^**^	0.148^**^	0.524^**^	0.504^**^	0.417^**^	1

**p<0.01.

### Hypothesis tests

This study first conducted hierarchical multiple regression analyses using SPSS to test Hypotheses H1, H2, H3a, H3b, H4a, and H4b, while providing preliminary assessment of mediating and moderating effects. Further applying Edwards and Lambert’s (2007) methodological framework, bootstrapping analysis with 5,000 resamples was employed to rigorously test the mediation effects, while the moderated mediation pathways were examined using Hayes’ PROCESS macro. Following Cohen et al.’s (2005) recommendations, interaction effect plots were constructed using digital literacy values at mean ±1 standard deviation to visually verify the moderating effects.

Hypothesis testing regarding the effects of digital technology requirements on employees’ proactive behavior (H1) and turnover intention (H2) revealed statistically significant positive effects. As evidenced in Table 3 (M2 and M10), requirements exerted a significant positive influence on proactive behavior (*β* = 0.547, *p* < 0.01) and similarly promoted turnover intention (*β* = 0.633, *p* < 0.01). These results empirically support both H1 and H2.

**Table 3:** Results for hierarchical regression analysis.

Independent variables	Dependent variables											
	Proactive behavior			Challenge stress			Turnover intention			Hindrance stress		
	M2	M3	M4	M6	M7	M8	M10	M11	M12	M14	M15	M16
Control												
Gender	−0.064	−0.031	−0.053	−0.078	−0.082	−0.076	−0.107	−0.076	−0.091	−0.039	−0.041	−0.044
Age	0.039	0.064	0.045	−0.034	−0.038	−0.033	0.028	0.041	0.027	0.004	0.002	0
Edu	0.136^*^	0.112	0.105	0.206^*^	0.193^*^	0.188*	0.082	0.132	0.089	−0.017	−0.024	−0.022
Dependent variables												
Digital technology requirements (DR)	0.547^***^		0.438^***^	0.722^***^	0.655^***^	0.693^***^	0.633^***^		0.407^***^	0.560^***^	0.525^***^	0.509^***^
Mediator												
Challenge stress		0.309^***^	0.152^***^									
Hindrance stress								0.535^***^	0.404^***^			
Moderator												
Digital literacy (DL)					0.211^***^	0.238^***^					0.112	0.101
Interaction												
DR*DL						0.329^***^						−0.139^*^
*R* ^2^	0.345	0.23	0.381	0.278	0.303	0.349	0.212	0.261	0.329	0.181	0.189	0.199
△*R*^2^	0.318	0.203	0.036	0.253	0.025	0.046	0.202	0.251	0.117	0.18	0.008	0.01
*F*	43.03	24.485	31.501	31.501	28.333	29.014	21.945	28.812	31.979	18.077	15.232	13.465
△*F*	158.591	86.421	19.065	114.728	11.584	22.903	83.837	111.044	57.063	71.814	3.335	3.944

*p<0.05, **p<0.01, ***p<0.001.

Mediation analysis for challenge stress in the digital technology requirements-proactive behavior linkage established: (1) M6 (Table 3) confirmed requirements’ significant positive effect on challenge stress (*β* = 0.722, *p* < 0.01), supporting H3a; (2) M3 demonstrated challenge stress’s significant positive impact on proactive behavior (*β* = 0.309, *p* < 0.01), validating H3b; (3) Comparative analysis of M2 and M4 revealed that while challenge stress partially mediated the relationship, digital requirements retained significant direct effects on proactive behavior (*β* = 0.438, *p* < 0.01), collectively evidencing partial mediation and preliminary confirmation of H3.

Following Edwards and Lambert’s (2007) recommendations, this study further applied bootstrapping (5,000 resamples) to verify the mediating effect of challenge stress, with specific results presented in Table 4. Table 4 data indicate that the indirect effect of digital technology requirements on proactive behavior via challenge stress is 0.109 (95% Bias-corrected CI [0.043, 0.179]), excluding zero with *p* < 0.05. This confirms a statistically significant mediating pathway. Furthermore, as neither the direct effect (95% CI [0.341, 0.535]) nor total effect (95% CI [0.462, 0.633]) confidence intervals contain zero, challenge stress demonstrates partial mediation, thereby further supporting Hypothesis H3.

**Table 4:** Bootstrap test results for direct effect and indirect effect of challenge stress in digital technology requirement and proactive behavior.

Path	Effect	se	LLCI	ULCI
Total	0.547	0.044	0.462	0.633
Direct	0.438	0.049	0.341	0.535
Indirect	0.109	0.035	0.043	0.179

Mediation analysis for hindrance stress in the digital technology requirements-turnover intention relationship established: (1) Model M14 (Table 3) confirmed requirements’ significant positive effect on hindrance stress (*β* = 0.560, *p* < 0.01), supporting H4a; (2) Model M11 demonstrated hindrance stress’s significant positive impact on turnover intention (*β* = 0.535, *p* < 0.01), validating H4b; (3) Comparative analysis of Models M10 and M12 revealed that while hindrance stress partially mediated the relationship, digital requirements retained significant direct effects on turnover intention (*β* = 0.407, *p* < 0.01), collectively evidencing partial mediation and preliminary confirmation of H4.

Consistent with Edwards and Lambert’s (2007) methodology, bootstrapping analysis (5,000 resamples) further validated the mediating role of hindrance stress, with results detailed in Table 5. The data indicate a significant indirect effect of digital technology requirements on turnover intention via hindrance stress (effect = 0.226, 95% Bias-corrected CI [0.133, 0.337]). This interval excludes zero (*p* < 0.05), confirming statistical significance. Furthermore, the exclusion of zero from 95% CIs for both direct and total effects establish partial mediation by hindrance stress, thereby fully supporting Hypothesis H4.

**Table 5:** Bootstrap test results for direct effect and indirect effect of hindrance stress in digital technology requirement and turnover intention.

Path	Effect	se	LLCI	ULCI
Total	0.633	0.069	0.497	0.769
Direct	0.407	0.071	0.268	0.546
Indirect	0.226	0.052	0.133	0.337

The moderating effect testing for digital literacy (H5a & H6a) involved mean-centering digital technology requirements, challenge stress, hindrance stress, and digital literacy, with interaction terms computed by multiplying standardized requirements and digital literacy scores to mitigate multicollinearity; hierarchical regression analyses designated challenge stress (M8) and hindrance stress (M16) as dependent variables, sequentially entering: (1) control variables (gender, age, education), (2) main effects (digital technology requirements + digital literacy), and (3) the Requirements × Literacy interaction term into models; Table 3 results (M8/M16) demonstrate the interaction term exerted a significant positive effect on challenge stress (*β* = 0.329, *p* < 0.01) while showing a significant negative effect on hindrance stress (*β* = −0.139, *p* < 0.05), indicating higher digital literacy strengthens the positive impact of digital requirements on challenge stress (supporting H5a) and weakens their impact on hindrance stress (supporting H6a).

**Figure 2:**
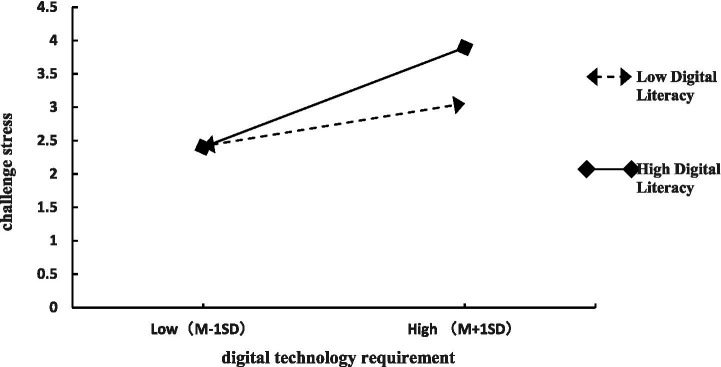
Interaction between digital technology requirement and digital literacy on challenge stress.

**Figure 3:**
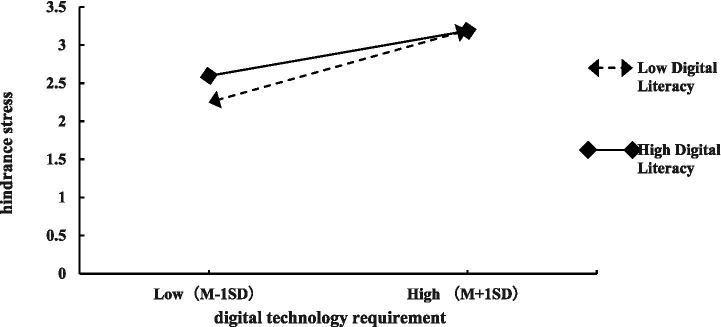
Interaction between digital technology requirement and digital literacy on hindrance stress.

To visually demonstrate digital literacy’s moderating effects, interaction effect plots were constructed using values at mean ±1 standard deviation of digital literacy, following Cohen et al.’s (2005) analytical guidelines, as presented in Figures 2, 3. Figure 2 visually demonstrates that under high digital literacy conditions (*Moderator + 1SD*), digital technology requirements exert a steeper positive slope on challenge stress; conversely, with low digital literacy (*Moderator − 1SD*), requirements yield a flatter slope in influencing challenge stress, thereby reconfirming partial support for Hypothesis H5a. Figure 3 reveals that under high digital literacy conditions (*Moderator + 1SD*), digital technology requirements exert a significantly attenuated positive effect on hindrance stress; whereas with low digital literacy (*Moderator − 1SD*), requirements demonstrate a substantially amplified effect on hindrance stress, thereby reconfirming partial support for Hypothesis H6a.

Moderated Mediation Testing (H5b & H6b): Further validation of the moderated mediation effects of digital literacy across dual pathways was conducted using Hayes’ PROCESS macro (Model 14), with statistical outcomes detailed in Tables 6, 7. For the challenge stress pathway, Table 6 indicates that under high digital literacy, the indirect effect of digital technology requirements on proactive behavior via challenge stress is significantly stronger than under low literacy conditions. Complementarily, Table 7 reveals a statistically significant moderated mediation index (Index = 0.05, 95% Bias-corrected CI [0.016, 0.092], excluding zero). This demonstrates differential moderation effects across literacy levels, with significantly amplified mediation under high digital literacy. Synthesized with prior analyses, these results empirically validate Hypothesis H5b. Regarding the hindrance stress path: Although the effect of enterprise digital technology requirements on employee turnover intention via hindrance stress was stronger under low levels of digital literacy than under high levels (Table 6), the index of moderated mediation included zero within its 95% bias-corrected confidence interval [−0.124, 0.002] (Table 7). This indicates that the mediation effect was consistent across different levels of digital literacy, signifying the absence of a moderated mediation effect. Consequently, Hypothesis H6b was not supported.

**Table 6:** The test of moderation mediation effect at different levels of digital literacy.

Mediator	Moderator	Levels	Effect	BootSE	BootLLCI	BootULCI
Challenge stress	Low	−0.847	0.063	0.026	0.019	0.119
	M	0.000	0.105	0.031	0.043	0.167
	High	0.847	0.147	0.043	0.061	0.232
Hindrance stress	Low	−0.847	0.253	0.054	0.157	0.366
	M	0.000	0.205	0.051	0.119	0.319
	High	0.847	0.158	0.062	0.054	0.295

**Table 7:** Index of moderated mediation.

Moderator	Mediator	Index	BootSE	BootLLCI	BootULCI
Digital literacy	Challenge stress	0.050	0.019	0.016	0.092
	Hindrance stress	−0.056	0.031	−0.124	0.002

## Discussion

### Conclusion

This study focuses on enterprise digital technology requirements within the digital economy context. Grounded in the Cognitive Appraisal Theory of Stress and integrating the Job JD-R Model with the COR Theory, it examines employees’ cognitive appraisal of such requirements as stressors, which leads to divergent outcomes: positive (proactive behavior) and negative (turnover intention). Furthermore, it investigates the moderating role of employees’ digital literacy across these dual pathways. Based on empirical data from a questionnaire survey, the key findings are as follows.

Firstly, enterprise digital technology requirements may lead to positive outcomes in the form of employee proactive behavior (Hypothesis H1), but may also trigger negative consequences such as turnover intention (Hypothesis H2).

Secondly, when appraising digital technology requirements as stressors, employees may develop either challenge stress appraisals (Hypothesis H3a) or hindrance stress appraisals (Hypothesis H4a).

Thirdly, such stressors can positively influence proactive behavior by promoting challenge stress appraisals (Hypothesis H3), while simultaneously increasing turnover intention through induced hindrance stress appraisals (Hypothesis H4).

Fourthly, employees’ digital literacy enhances their positive appraisal of enterprise digital technology requirements as stressors while mitigating negative appraisals. Specifically, digital literacy strengthens the positive effect of digital technology requirements on challenge stress appraisals (H5a), but attenuates their effect on hindrance stress appraisals (H6a). Furthermore, digital literacy amplifies the positive indirect effect of digital technology requirements on proactive behavior through challenge stress (H5b), whereas its moderating effect on the pathway linking digital technology requirements to turnover intention via hindrance stress is non-significant (contradicting H6b). Two underlying mechanisms may explain this phenomenon: Firstly, the detrimental effects of stress demonstrate universality. The fundamental pathways linking hindrance stress appraisals to turnover intention—including induced frustration, perceived goal blockage, resource depletion, diminished job satisfaction, and reduced organizational commitment-operate irrespective of digital literacy levels. Even among digitally literate employees, intense hindrance stress arising from other sources (e.g., role ambiguity, interpersonal conflict, or perceived injustice) or residual stress after partial alleviation may still trigger turnover intentions through these inherent negative psychological mechanisms. Secondly, turnover decisions are inherently multi-dimensional. Turnover intention stems from a constellation of factors (job content, leadership, colleagues, compensation, career development, work-life balance, etc.). While digital literacy primarily facilitates coping with technological challenges, it proves insufficient to buffer the negative impact of hindrance stress on overall job evaluation (satisfaction and commitment)-critical antecedents of turnover. Once stress erodes these core attitudes, the literacy-turnover linkage becomes resistant to mitigation.

### Theoretical implications

This study advances the dual-path framework of enterprise digital technology implementation by elucidating how digital literacy-as a personal resource-shapes divergent outcomes stemming from organizational digital demands. Furthermore, it extends the application of the Cognitive Appraisal Theory of Stress to digital contexts through empirical validation.

Firstly, this study significantly advances the dual-path framework of enterprise digital technology implementation. Deeply resonating with Verhoef et al.’s (2021) proposition regarding the dual nature of digital technologies-simultaneously enabling value creation and incurring potential risks-we innovatively integrate a “Technology-Individual-Behavior” (TIB) analytical framework comprising *technology stressors*, *individual cognitive appraisals*, and *divergent behavioral outcomes*. This integration allows us to construct and empirically validate a comprehensive dual-path cognitive-behavioral model. The model elucidates how enterprise digital technology requirements, as core stressors, trigger two parallel yet independent cognitive pathways: (1) challenge stress appraisals ultimately fostering proactive behaviors (e.g., innovating, learning, problem-solving), and (2) hindrance stress appraisals culminating in turnover intention. This provides a theoretically grounded account for the differential manifestation of digital technology’s dual effects at the micro-individual level through cognitive-psychological mechanisms.

Secondly, this study directly addresses Tan et al.’s (2025) call for exploring boundary conditions of employee adaptation mechanisms in the AI era. By empirically testing the critical moderating role of digital literacy within the “stressor-appraisal-behavior” framework, we significantly extend boundary condition theories regarding how digital technologies influence employee responses. Our research establishes digital literacy-a multidimensional competence system encompassing technical knowledge, application skills, and adaptive attitudes-as a core boundary condition explaining differential employee reactions to technostress. Crucially, we reveal its *asymmetric moderating mechanism*: Digital literacy amplifies the positive impact of digital technology requirements on challenge stress appraisals (H5a) while attenuating their effect on hindrance stress appraisals (H6a), thereby optimizing the positive behavioral pathway. However, it fails to significantly moderate the hindrance stress → turnover intention pathway (H6b), highlighting the efficacy boundaries of individual capability resources across distinct stress transmission stages (cognitive appraisal vs. behavioral consequence). These findings yield pivotal micro-level evidence for optimizing human-AI coexistence paradigms. They challenge the prevailing cognitive framework of over-reliance on skill enhancement to address technological challenges, instead advocating for integrated organizational interventions to manage structural resilience in stress transmission pathways.

Finally, this study substantially advances the theoretical depth of the Cognitive Appraisal Theory of Stress in digital contexts by systematically unpacking the cognitive differentiation mechanisms through which enterprise digital technology requirements trigger dual-path appraisals (challenge vs. hindrance stress). Our empirical verification of technostressors driving dichotomous outcomes-proactive behavior versus turnover intention through differentiated appraisals-not only confirms the theory’s generalizability to digital workplaces but also pioneers the identification of digital literacy’s stage-specific moderation during primary appraisal (stressor → cognition). Consequently, we evolve traditional stress theory from a static “stimulus–response” framework into a dynamic “Technostress-Literacy Modulation-Cognitive Differentiation-Behavioral Polarization” (TLCD-BP) model, establishing a comprehensive theoretical anchor for explaining heterogeneous employee stress responses during digital transformation.

### Practical implications

Firstly, organizations must proactively institutionalize systematic digital literacy development programs by implementing preemptive training-covering technical operations, ethical decision-making, and stress management through simulations and case-based workshops-prior to deploying new technologies (e.g., AI systems) to disrupt hindrance stress formation at its source, while concurrently integrating digital literacy metrics into role assignments and career advancement pathways to incentivize self-driven competency enhancement.

Secondly, managers should implement differentiated strategies based on employees’ digital literacy levels: For high-literacy employees, amplify challenge appraisals through *technology-task fit* designs (e.g., granting AI customization rights, opening API access) to catalyze proactive behaviors; for low-literacy cohorts, initiate interventions including streamlined operations, obstacle reduction, psychological safety mechanisms, and technophobia mitigation.

Thirdly, HR managers must implement *precision-focused* and *preemptive* turnover intervention systems by: (1) constructing hindrance stress early-warning models through behavioral trace analytics (e.g., login latency rates, task failure frequency, training engagement metrics) to trigger timely interventions (e.g., dynamic workload modulation or personalized upskilling) for high-risk employees; and (2) deploying compensatory resource bundles (leader-member exchange enhancement, peer empowerment cohorts, benefit portfolio optimization) to mitigate turnover intention among employees experiencing entrenched hindrance stress-given our finding that digital literacy augmentation fails to attenuate established stress-turnover pathways.

### Limitations and directions of future research

Firstly, regarding research design, while this study primarily employed cross-sectional surveys for empirical analysis, future research should adopt Experience Sampling Methodology (ESM) to track employees’ stress appraisals and behavioral evolution across technology deployment phases (implementation → adaptation → proficiency), thereby mapping dynamic stress-behavior trajectories.

Secondly, to put it more rigorously, according to the Cognitive Appraisal Theory of Stress and Self-Efficacy Theory, resources (DL) generally influence employees’ appraisal through their belief in their own coping capabilities, namely digital self-efficacy (Aesaert et al., 2017; Ulfert-Blank and Schmidt, 2022). Therefore, digital self-efficacy should be incorporated into the model in future research.

Thirdly, concerning moderator selection, while this study focused on individual-level digital literacy as a boundary condition, future research should develop *cross-level moderated models* by integrating organizational- (e.g., digital transformation strategy archetypes), team- (e.g., human-AI collaboration intensity), and individual-level variables (e.g., learning agility), thereby constructing a multilevel moderation framework for technostress transmission.

Fourthly, regarding the sample size, the final dataset comprised 332 valid responses. This limitation is attributable to two primary factors: firstly, the inherent constraint of targeting only employees who have undergone or are currently undergoing digital transformation; and secondly, sample attrition resulting from the three-wave survey design. Although a sample size of 332 is statistically acceptable, its generalizability to the vast and diverse Chinese labor force may be limited.

Lastly, future research should conduct *cross-cultural comparative analyses* by sampling employees from divergent cultural contexts-contrasting individualism-oriented societies (e.g., United States) with collectivism-dominant cultures (e.g., Japan)-to examine cultural variations in digital literacy’s moderation effect sizes on stress appraisals, thereby revealing the underlying moderating mechanisms of cultural value dimensions.

## Data Availability

The original contributions presented in the study are included in the article/supplementary material, further inquiries can be directed to the corresponding author.

## Appendix

**Table A1:** Means and standard deviation of digital technology requirements.

Items	*M*	SD
1. TR1 Acquiring important digital technologies.	3.9	0.855
2. TR2 Identifying new digital opportunities.	3.84	0.990
3. TR3 Responding to digital transformation	3.99	1.013
4. TR4 Mastering the state-of-the-art digital technologies	3.85	0.913
5. TR5 Developing innovative products/service/process using digital technology	3.95	0.985

## References

[ref1] Aesaert K. Voogt J. Kuiper E. Van Braak J. (2017). Accuracy and bias of ICT self-efficacy: an empirical study into students’ over- and underestimation of their ICT competences. Comput. Human Behav. 75, 92–102. doi: 10.1016/j.chb.2017.05.010

[ref2] Azharudeen N. T. Arulrajah A. A. (2018). The relationships among emotional demand, job demand, emotional exhaustion and turnover intention. Int. Bus. Res. 11:8. doi: 10.5539/ibr.v11n11p8

[ref3] Bakker A. B. Demerouti E. Verbeke W. (2004). Using the job demands-resources model to predict burnout and performance. Hum. Resour. Manag. 43, 83–104. doi: 10.1002/hrm.20004

[ref4] Bandura A. (1989). Regulation of cognitive processes through perceived self-efficacy. Dev. Psychol. 25, 729–735. doi: 10.1037/0012-1649.25.5.729

[ref5] Bankins S. Ocampo A. C. Marrone M. Restubog S. L. D. Woo S. E. (2024). A multilevel review of artificial intelligence in organizations: implications for organizational behavior research and practice. J. Organ. Behav. 45, 159–182. doi: 10.1002/job.2735

[ref6] Boswell W. R. Olson-Buchanan J. B. LePine M. A. (2004). Relations between stress and work outcomes: the role of felt challenge, job control, and psychological strain. J. Vocat. Behav. 64, 165–181. doi: 10.1016/S0001-8791(03)00049-6

[ref7] Brougham D. Haar J. (2020). Technological disruption and employment: the influence on job insecurity and turnover intentions: a multi-country study. Technol. Forecast. Soc. Change 161:120276. doi: 10.1016/j.techfore.2020.120276

[ref8] Cavanaugh M. A. Boswell W. R. Roehling M. V. Boudreau J. W. (2000). An empirical examination of self-reported work stress among U.S. managers. J. Appl. Psychol. 85, 65–74. doi: 10.1037/0021-9010.85.1.65, 10740957

[ref9] Cheng B. Lin H. Kong Y. (2023). Challenge or hindrance? How and when organizational artificial intelligence adoption influences employee job crafting. J. Bus. Res. 164:113987. doi: 10.1016/j.jbusres.2023.113987

[ref9001] China Academy of Information and Communications Technology (2024). China digital economy development research report.

[ref10] Cohen J. Cohen P. West S. G. Aiken L. S. Dodhia R. M. (2005). Book review-a review of applied multiple regression/correlation analysis for the behavioral sciences. J. Educ. Behav. Stat. 30:227. doi: 10.1177/019394598000200320

[ref11] Crawford E. R. LePine J. A. Rich B. L. (2010). Linking job demands and resources to employee engagement and burnout: a theoretical extension and meta-analytic test. J. Appl. Psychol. 95, 834–848. doi: 10.1037/a0019364, 20836586

[ref12] Demerouti E. Bakker A. B. Nachreiner F. Schaufeli W. B. (2001). The job demands-resources model of burnout. J. Appl. Psychol. 86, 499–512. doi: 10.1037/0021-9010.86.3.499, 11419809

[ref13] Du L. Z. Chen Y. X. (2023). Does time stressor promote or inhibit employees' proactive work behavior? Cognitive appraisals of stress as mediator and time management skills as moderator. Hum. Resour. Dev. China 40, 6–20. doi: 10.16471/j.cnki.11-2822/c.2023.4.001

[ref14] Edwards J. R. Lambert L. S. (2007). Methods for integrating moderation and mediation: a general analytical framework using moderated path analysis. Psychol. Methods 12, 1–22. doi: 10.1037/1082-989X.12.1.1, 17402809

[ref15] Fu F. Q. Zha W. H. Zhou Q. W. (2024). The influence of digital technology induced job requirements on employee innovative behavior: a study based on paradox theory. Hum. Resour. Dev. China 41, 69–83. doi: 10.16471/j.cnki.11-2822/c.2024.3.005

[ref16] Glikson E. Woolley A. W. (2020). Human trust in artificial intelligence: review of empirical research. Acad. Manage. Ann. 14, 627–660. doi: 10.5465/annals.2018.0057, 41370688

[ref17] Gonzalez-Mulé E. Kim M. M. Ryu J. W. (2021). A meta-analytic test of multiplicative and additive models of job demands, resources, and stress. J. Appl. Psychol. 106, 1391–1411. doi: 10.1037/apl0000840, 32955269

[ref18] Griffin M. A. Neal A. Parker S. K. (2007). A new model of work role performance: positive behavior in uncertain and interdependent contexts. Acad. Manag. J. 50, 327–347. doi: 10.5465/amj.2007.24634438

[ref19] Guo D. J. Wu M. Y. (2024). How does digital technology application affect job insecurity? Soc. Sci. United Front. 9, 106–118.

[ref20] Halbesleben J. R. B. Neveu J.-P. Paustian-Underdahl S. C. Westman M. (2014). Getting to the “COR”: understanding the role of resources in conservation of resources theory. J. Manage. 40, 1334–1364.

[ref21] Harman H. H. (1976). Modern factor analysis. Chicago and London: University of Chicago Press.

[ref22] He Q. Liu M. Z. Li X. Y. (2024). Will artificial intelligence trigger employees' knowledge hiding? A relative deprivation theory perspective. Foreign Econ. Manag. 46, 55–70. doi: 10.16538/j.cnki.fem.20231226.101

[ref23] He C. Teng R. Song J. (2024). Linking employees' challenge-hindrance appraisals toward AI to service performance: the influences of job crafting, job insecurity and AI knowledge. Int. J. Contemp. Hosp. Manage. 36, 975–994. doi: 10.1108/ijchm-07-2022-0848

[ref24] Huth K. B. S. Chung-Yan G. A. (2022). Quantifying the evidence for the absence of the job demands and job control interaction on workers' well-being: a Bayesian meta-analysis. J. Appl. Psychol. 108, 1060–1072. doi: 10.1037/apl0001066, 36442031

[ref25] Ingusci E. Signore F. Giancaspro M. L. Manuti A. Molino M. Russo V. . (2021). Workload, techno overload, and behavioral stress during COVID-19 emergency: the role of job crafting in remote workers. Front. Psychol. 12:655148. doi: 10.3389/fpsyg.2021.655148, 33912116 PMC8072041

[ref26] Jarrahi M. H. (2018). Artificial intelligence and the future of work: human-AI symbiosis in organizational decision making. Bus. Horiz. 61, 577–586. doi: 10.1016/j.bushor.2018.03.007

[ref27] Jia N. Luo X. Fang Z. Liao C. (2024). When and how artificial intelligence augments employee creativity. Acad. Manag. J. 67, 5–32. doi: 10.5465/amj.2022.0426

[ref28] Jianwu J. Hanhuan L. Jieyu H. (2024). A meta-analysis of the impact of AI application on employees in the workplace. Adv. Psychol. Sci. 32, 1621–1637. doi: 10.3724/SP.J.1042.2024.01621

[ref29] Johnson A. Dey S. Nguyen H. Groth M. Joyce S. Tan L. . (2020). A review and agenda for examining how technology-driven changes at work will impact workplace mental health and employee well-being. Aust. J. Manage. 45, 402–424. doi: 10.1177/0312896220922292

[ref30] Kelloway E. K. Gottlieb B. H. Barham L. (1999). The source, nature, and direction of work and family conflict: a longitudinal investigation. J. Occup. Health Psychol. 4, 337–346. doi: 10.1037/1076-8998.4.4.337, 10526838

[ref31] Khin S. Ho T. C. (2019). Digital technology, digital capability and organizational performance: a mediating role of digital innovation. Int. J. Innov. Sci. 11, 177–195. doi: 10.1108/IJIS-08-2018-0083

[ref32] Kong L. Li X. Y. (2019). Research on the influence mechanism of leader rejection and colleague rejection on employee initiative behavior. J. Tech. Econ. Manag. 5, 68–73.

[ref33] Kożusznik M. W. Peiró J. M. Soriano A. Navarro Escudero M. (2018). Out of sight, out of mind? Environ. Behav. 50, 86–115. doi: 10.1177/0013916517691323

[ref34] Lazarus R. S. Folkman S. (1984). Stress, appraisal, and coping. New York: Springer Publishing Company.

[ref35] Lazarus R. S. Folkman S. (1987). Transactional theory and research on emotions and coping. Eur. J. Personal. 1, 141–169. doi: 10.1002/per.2410010304

[ref36] LePine J. A. Podsakoff N. P. LePine M. A. (2005). A meta-analytic test of the challenge stressor–hindrance stressor framework: an explanation for inconsistent relationships among stressors and performance. Acad. Manag. J. 48, 764–775. doi: 10.5465/amj.2005.18803921

[ref37] Li J. J. Bonn M. A. Ye B. H. (2019). Hotel employee's artificial intelligence and robotics awareness and its impact on turnover intention: the moderating roles of perceived organizational support and competitive psychological climate. Tour. Manag. 73, 172–181. doi: 10.1016/j.tourman.2019.02.006

[ref38] Li X. M. Chen L. (2024). The impact mechanism of human-AI interaction on innovative work behavior: evidence from smart power plants. Foreign Econ. Manag. 46, 105–120. doi: 10.16538/j.cnki.fem.20240907.102

[ref39] Li X. J. Chen Z. Xia X. L. (2022). The impact of digital literacy on farmers' entrepreneurial behavior: an analysis based on the spatial Durbin model. J. Zhongnan Univ. Econ. Law 1, 123–134. doi: 10.19639/j.cnki.issn1003-5230.2022.0006

[ref40] Li H. Guo C. H. (2025). "Open books bring benefits" or "closed books hide treasures"? The double-edged sword effect of algorithm opacity on employee creativity. Nankai Bus. Rev. Advance online publication. doi: 10.3969/j.issn.1008-3448.2025.01.001

[ref41] Li Z. B. Li R. (2013). A review of the literature of challenge and hindrance stressor. Foreign Econ. Manag. 35, 40–49. doi: 10.16538/j.cnki.fem.2013.05.008

[ref42] Liang X. Guo G. Shu L. Gong Q. Luo P. (2022). Investigating the double-edged sword effect of AI awareness on employee's service innovative behavior. Tour. Manag. 92:104564. doi: 10.1016/j.tourman.2022.104564

[ref43] Liu S. Cheng P. (2025). The influence mechanism of enterprise digitalization on employee taking charge. Int. J. Manpow. 46, 556–572. doi: 10.1108/IJM-05-2024-0316

[ref44] Liu Y. S. Liu Y. Y. Zhang F. Chu F. L. (2024). Threat or challenge: the double-edged sword effect of artificial intelligence usage on employee innovation performance. Collect. Essays Financ. Econ. 9, 91–102. doi: 10.13762/j.cnki.cjlc.2024.09.003

[ref45] Loureiro S. M. C. Bilro R. G. Neto D. (2023). Working with AI: can stress bring happiness? Serv. Bus. 17, 233–255. doi: 10.1007/s11628-022-00514-8

[ref46] Ma R. (2024). The impact effects of enterprise digital technology application: review and prospects. Sci. Decis. Making 5, 265–279.

[ref47] Ma L. Li S. R. (2025). Artificial intelligence anxiety and innovative behavior of new generation employees: the roles of organizational attachment and job crafting. Sci. Technol. Prog. Policy 42, 132–140. doi: 10.6049/kjjbydc.2024070209

[ref48] Madsen S. R. Miller D. John C. R. (2005). Readiness for organizational change: do organizational commitment and social relationships in the workplace make a difference? Hum. Resour. Dev. Q. 16, 213–234. doi: 10.1002/hrdq.1134

[ref49] Makowska-Tłomak E. Bedyńska S. Skorupska K. Nielek R. Kornacka M. Kopeć W. (2023). Measuring digital transformation stress at the workplace–development and validation of the digital transformation stress scale. PLoS One 18:e0287223. doi: 10.1371/journal.pone.0287223, 37851687 PMC10584111

[ref50] Makowska-Tłomak E. Bedyńska S. Skorupska K. Paluch J. (2022). Blended online intervention to reduce digital transformation stress by enhancing employees’ resources in COVID-19. Front. Psychol. 13:732301. doi: 10.3389/fpsyg.2022.732301, 35391985 PMC8982670

[ref51] Malhotra N. K. Kim S. S. Patil A. (2006). Common method variance in IS research: a comparison of alternative approaches and a reanalysis of past research. Manag. Sci. 52, 1865–1883. doi: 10.1287/mnsc.1060.0597, 19642375

[ref52] Matarazzo M. Penco L. Profumo G. Quaglia R. (2021). Digital transformation and customer value creation in made in Italy SMEs: a dynamic capabilities perspective. J. Bus. Res. 123, 642–656. doi: 10.1016/j.jbusres.2020.10.033

[ref53] May D. R. Gilson R. L. Harter L. M. (2004). The psychological conditions of meaningfulness, safety and availability and the engagement of the human spirit at work. J. Occup. Organ. Psychol. 77, 11–37. doi: 10.1348/096317904322915892

[ref54] Meng X. J. Xu P. (2024). Impact of AI awareness on job burnout: based on survey data from technology companies. Res. Econ. Manag. 45, 99–110. doi: 10.13502/j.cnki.issn1000-7636.2024.10.006

[ref55] Mueller S. L. Thomas A. S. (2001). Culture and entrepreneurial potential: a nine country study of locus of control and innovativeness. J. Bus. Venturing 16, 51–75. doi: 10.1016/S0883-9026(99)00039-7

[ref56] Ng W. (2012). Can we teach digital natives digital literacy? Comput. Educ. 59, 1065–1078. doi: 10.1016/j.compedu.2012.04.016

[ref57] Ouyang C. H. Zhu Y. Y. (2025). Workplace digitalisation and employee creativity: the role of autonomous motivation and career agility. Soft Sci. doi: 10.13956/j.ss.1001-8409.2025.07.03

[ref58] Park Y. Liu Y. Headrick L. (2020). When work is wanted after hours: testing weekly stress of information communication technology demands using boundary theory. J. Organ. Behav. 41, 518–534. doi: 10.1002/job.2461

[ref59] Parker S. K. Collins C. G. (2010). Taking stock: integrating and differentiating multiple proactive behaviors. J. Manage. 36, 633–662. doi: 10.1177/0149206308321554

[ref60] Piccinini E. Gregory R. W. Kolbe L. M. (2015). “Changes in the producer-consumer relationship - towards digital transformation” in Wirtschaftsinformatik 2015 proceedings. Wiesbaden: Springer Vieweg.

[ref61] Podsakoff P. M. MacKenzie S. B. Lee J.-Y. Podsakoff N. P. (2003). Common method biases in behavioral research: a critical review of the literature and recommended remedies. J. Appl. Psychol. 88, 879–903. doi: 10.1037/0021-9010.88.5.879, 14516251

[ref62] Presbitero A. Teng-Calleja M. (2023). Job attitudes and career behaviors relating to employees' perceived incorporation of artificial intelligence in the workplace: a career self-management perspective. Pers. Rev. 52, 1169–1187. doi: 10.1108/pr-02-2021-0103

[ref63] Raj M. Seamans R. (2019). Primer on artificial intelligence and robotics. J. Organ. Des. 8, 1–14. doi: 10.1186/s41469-019-0050-0

[ref64] Richard B. (1970). Back-translation for cross-cultural research. J. Cross-Cult. Psychol. 1, 185–216.

[ref65] Smith C. Smith J. B. Shaw E. (2017). Embracing digital networks: entrepreneurs' social capital online. J. Bus. Ventur. 32, 18–34. doi: 10.1016/j.jbusvent.2016.10.003

[ref66] Su L. L. Zhang H. Y. Peng Y. L. (2021). Research on the mechanism of farmers' digital literacy driving digital rural development. E-Government 10*, 42–56. doi: 10.16582/j.cnki.dzzw.2021.10.004

[ref9002] Tan M. Yin X. Zheng G. Xiong P. (2025). Workplace artificial intelligence role classification: Impacts on employee psychology and behavior and coping strategies. Adv. Psychol. Sci., 33, 933–947. doi: 10.3724/SP.J.1042.2025.0933

[ref67] Tang P. M. Koopman J. Mai K. M. (2023). No person is an island: unpacking the work and after-work consequences of interacting with artificial intelligence. J. Appl. Psychol. 108, 1766–1789. doi: 10.1037/apl0001103, 37307359

[ref68] Tarafdar M. Cooper C. L. Stich J. (2019). The technostress trifecta - techno eustress, techno distress and design: theoretical directions and an agenda for research. Inf. Syst. J. 29, 6–42. doi: 10.1111/isj.12169

[ref69] Tarafdar M. Pirkkalainen H. Salo M. Makkonen M. (2020). Taking on the “dark side”––coping with technostress. IT Professional 22, 82–89. doi: 10.1109/MITP.2020.2977343

[ref70] Teng R. Zhou S. Zheng W. Ma C. (2024). Artificial intelligence (AI) awareness and work withdrawal: evaluating chained mediation through negative work-related rumination and emotional exhaustion. Int. J. Contemp. Hosp. Manag. 36, 2311–2326. doi: 10.1108/ijchm-02-2023-0240

[ref71] Tett R. P. Meyer J. P. (1993). Job satisfaction, organizational commitment, turnover intention, and turnover: path analyses based on meta-analytic findings. Pers. Psychol. 46, 259–293. doi: 10.1111/j.1744-6570.1993.tb00874.x

[ref72] Tims M. Bakker A. B. Derks D. (2012). Development and validation of the job crafting scale. J. Vocat. Behav. 80, 173–186. doi: 10.1016/j.jvb.2011.05.009

[ref73] Tong S. Jia N. Luo X. Fang Z. (2021). The Janus face of artificial intelligence feedback: deployment versus disclosure effects on employee performance. Strateg. Manage. J. 42, 1600–1631. doi: 10.1002/smj.3322

[ref74] Ulfert-Blank A.-S. Schmidt I. (2022). Assessing digital self-efficacy: review and scale development. Comput. Educ. 191:104626. doi: 10.1016/j.compedu.2022.104626

[ref75] Van Den Broeck A. De Cuyper N. De Witte H. Vansteenkiste M. (2010). Not all job demands are equal: differentiating job hindrances and job challenges in the job demands-resources model. Eur. J. Work Organ. Psychol. 19, 735–759. doi: 10.1080/13594320903223839

[ref76] Verma S. Singh V. (2022). Impact of artificial intelligence-enabled job characteristics and perceived substitution crisis on innovative work behavior of employees from high-tech firms. Comput. Hum. Behav. 131:107215. doi: 10.1016/j.chb.2022.107215

[ref9003] Verhoef P. C. Broekhuizen T. Bart Y. Bhattacharya A. Qi Dong J. Fabian N. . (2021). Digital transformation: A multidisciplinary reflection and research agenda. J. Bus. Res., 122:889–901. doi: 10.1016/j.jbusres.2019.09.022, 33912116

[ref77] Wang H. H. Du M. (2021). Digital technology, employee participation and enterprise innovation performance. R D Manage. 33, 138–148. doi: 10.13581/j.cnki.rdm.20200757

[ref78] Wang W. Liu K. X. (2025). Research on the dual effects of artificial intelligence (AI) technology application on employees' knowledge behaviors from a cognitive appraisal perspective. Sci. Technol. Manag. Res. 45, 111–120. doi: 10.3969/j.issn.1000-7695.2025.16.012

[ref79] Wang T. Zhan X. J. Yu W. (2024). The influence of AI awareness on employee's psychological and behavioral outcomes and its theoretical explanation. Adv. Psychol. Sci. 32, 1195–1213. doi: 10.3724/SP.J.1042.2024.01195, 41207781

[ref80] Wang H. Zhang H. Chen Z. Zhu J. Zhang Y. (2022). Influence of artificial intelligence and robotics awareness on employee creativity in the hotel industry. Front. Psychol. 13:834160. doi: 10.3389/fpsyg.2022.834160, 35300168 PMC8922015

[ref81] Wei H. M. Pan Q. Q. (2012). Research on employee proactive behavior and its drivers in complex environments. Ent. Econ. 3, 94–97. doi: 10.13529/j.cnki.enterprise.economy.2012.03.036

[ref82] Xu G. L. (2023). The impact of artificial intelligence anxiety on employee deviant behavior: a moderated mediation model. East China Econ. Manag. 37, 120–128. doi: 10.19629/j.cnki.34-1014/f.221129012

[ref83] Xu Y. Zhao D. D. Zhang R. J. Liu X. J. (2024). A study on the inverted U-shaped relationship between boundarylessness and employee proactive behavior in distributed office. Hum. Resour. Dev. China 41, 6–20. doi: 10.16471/j.cnki.11-2822/c.2024.1.001

[ref84] Yu S. X. Liu M. Liu S. S. Liu T. T. (2024). Research on the influence mechanism of algorithmic control on the gig workers' turnover intention. Chin. J. Manage. 21, 1152–1162. doi: 10.3969/j.issn.1672-884x.2024.08.005

[ref85] Zhang H. Gao Z. H. Li H. L. (2023). Gain or loss? The double-edged sword effect of artificial intelligence usage on innovation behavior. Sci. Technol. Prog. Policy 40, 1–11. doi: 10.6049/kjjbydc.2023030249

[ref86] Zhang Y. Zhang Y. Ng T. W. H. Lam S. S. K. (2019). Promotion- and prevention-focused coping: a meta-analytic examination of regulatory strategies in the work stress process. J. Appl. Psychol. 104, 1296–1323. doi: 10.1037/apl0000404, 30945878

[ref87] Zhao L. Zhai X. Y. (2018). The influence of work autonomy on voice behavior: the roles of work engagement and proactive personality. J. Grad. School Chinese Acad. Soc. Sci. 6, 33–44.

[ref88] Zhou W. W. Gu X. X. Li W. L. Wang Q. Ding G. F. (2023). Why can job crafting be a strategy for employees to cope with abusive supervision? Based on the transactional theory of stress. Hum. Resour. Dev. China 40, 21–34. doi: 10.16471/j.cnki.11-2822/c.2023.4.002

[ref89] Zhu X. M. Ren J. J. He Q. (2020). Will the application of artificial intelligence technology trigger employees' negative emotions? Based on the perspective of resource conservation theory. Chin. J. Clin. Psychol. 28, 1285–1288. doi: 10.16128/j.cnki.1005-3611.2020.06.042

[ref90] Zou Y. Zhou Y. R. Huang Q. X. (2023). Research on proactive learning behavior of employees under the impact of artificial intelligence technology. Sci. Technol. Manag. Res. 16, 180–187. doi: 10.3969/j.issn.1000-7695.2023.17.021

